# Major colorectal surgery with Hugo™ RAS: initial experience of a German center and a review of the literature

**DOI:** 10.1007/s13304-024-01939-8

**Published:** 2024-07-09

**Authors:** Orlin Belyaev, Tim Fahlbusch, Illya Slobodkin, Wademar Uhl

**Affiliations:** Department of General and Visceral Surgery, St. Josef University Hospital, Ruhr University Bochum, Gudrunstr. 56, 44791 Bochum, Germany

**Keywords:** Robotic assisted, Hugo, Colorectal surgery, Setup, Total mesorectal excision

## Abstract

The recently introduced Hugo RAS robotic platform has mostly been used for well standardized urologic and gynaecologic procedures. Experience with this new system in general surgery and especially in major colorectal surgery is very limited. This is a retrospective series of the first 25 consecutive non-selected colorectal surgeries performed at a single German center. The lessons learned from our initial experience are presented along with a systematic review of the currently available literature on this topic. Ten sigmoid and seven rectal resections, four right and one left hemicolectomies, two Hartmann’s reversals and an abdominoperineal resection were performed in 14 women and 11 men at the median age of 66 years for 12 benign findings and 13 malignancies. All procedures were performed using four robotic ports and a single 12 mm assistant port. Median docking, console and total operative times were 12, 170 and 270 min. Median blood loss was < 100 ml, and median stay was 8 days. The literature review identified five case series with a total of 23 colorectal procedures: 9 right and 1 left hemicolectomies, 5 ileocaecal, and 4 rectal and 4 sigmoid resections. Results corresponded to ours despite variations in setup used by different authors. A wide spectrum of major colorectal surgery can be safely and effectively performed with the Hugo RAS, even in a cohort of non-selected patients. Ongoing software and hardware upgrade, introduction of robotic energy devices and increasing surgical experience are expected to facilitate procedures and reduce duration of surgery.

## Introduction

The Hugo™ RAS (Medtronic, Dublin, Ireland) platform is a much-awaited alternative to the da Vinci robotic system, which received its European certification for use in gynaecologic and urologic surgery in early 2022 and for general surgery later the same year. It consists of a system tower, an open console, including a widescreen HD-3D display with dedicated glasses, two pistol-like handgrips as arm-controllers and a footswitch panel, as well as four independent arm carts with a wide manoeuvre range. So far, about 60 Hugo™ RAS devices have been installed in Europe and mainly been used in performing common and well-standardized urologic and gynaecologic procedures such as prostatectomy, nephrectomy and hysterectomy [1–3]. In contrast, experience with Hugo in general surgery has been limited to minor procedures such as cholecystectomy, bariatric procedures and hernia repair [4–7]. Especially, reports on major colorectal resections are very few and represented only by case reports and small case series [7–11].

The aim of this report was to demonstrate the feasibility and safety of the Hugo RAS platform for major colorectal surgery in a non-selected patient group and to summarize current experience through a systematic review of existent literature. The focus of the report lies on tips and tricks in the setup of the platform to allow fluent and safe surgical workflow.

## Methods

### Patients and arrangements

A retrospective chart review was performed of the first 25 patients who underwent robotic-assisted colorectal procedures at our hospital after beginning work with the Hugo RAS in February 2023 till March 2024. Due to regulatory issues in Germany, no procedures were performed between April and October 2023. No specific exclusion criteria were applied for patient selection. All patients gave written informed consent for robotic surgery and were personally informed by the operating surgeon about the procedure in all its aspects, including its novelty and limited experience with this system. They signed a special informed consent including additional remarks about possible device failures and device-induced complications. This study was approved by the Ethics Committee of the Ruhr University Bochum (Nr. 23-7872-BR). It was conducted in accordance with the Declaration of Helsinki. In addition to clinical perioperative parameters, the exact data on trocar positioning, docking and console times as well as technical performance of the device were gathered. All surgeries were recorded by the DS1 system of the platform and were reviewed via the Touch Surgery™ application. Docking time was defined as the time needed to attach and test all manipulator arms to the instruments after trocar placement. Console time was the time between docking and final undocking. Total operative time was defined as time from skin incision till skin closure.

The surgical team had previous extensive experience with open and laparoscopic, but no clinical experience with robotic colorectal surgery. They completed theoretical and practical training offered by the vendor as already described in a previous report by us [4]. All procedures described in this case series were performed by the same surgeon (O.B.) System components of the Hugo RAS platform are repeatedly described in detail elsewhere [4–8].

### Surgical technique, patient positioning, and trocar placement

The first cases were done according to the available setup guides of the manufacturer. Due to limited exposure of the surgical field and arm collisions, different variations in the position of trocars and settings of the robotic arms were tested until a comfortable optimal setup was established for every type of procedure. In summary all four arms of Hugo and an additional 12 mm laparoscopic port were used in every patient. No AirSeal® system was used. The standard set of robotic instruments included monopolar shears, a bipolar grasper, and a double-fenestrated grasper. A cadiere and a large needle holder were used on demand. The 0° optic was used as a standard, while the 30° optic was rarely necessary. Irrigation, suction, clip application, stapling and insertion of suture material was applied through the assistant port. The abdomen was always entered via a mini-laparotomy, gas insufflator attached to the fourth arm, and the camera held by hand and mounted to its arm last to facilitate visualisation of instruments and allow their safe and fast docking.

## Results

The first robotic-assisted colorectal surgery with the Hugo™ RAS was a sigmoidectomy performed by our team on February 13th, 2023. It was at the same time the first-in-human colorectal procedure with the Hugo™ RAS was performed in Germany. Patients’ characteristics and outcomes are presented in chronological order of performed procedures in Tables 1 and 2.

**Table 1:** Patients’ characteristics of 25 colorectal procedures in chronological order

Nr	Sex	Age	BMI	Comorbidities	Previous surgery	Diagnosis	Surgery	Concomitant procedures	Anastomosis
1	F	65	27	AH, breast cancer	Open HE + OE, CCE, AE	Diverticulitis, adhesions	SR	Adhesiolysis	33 CEEA at 14 cm
2	M	72	19	Stroke, DM		Rectal cancer at 5 cm after TNT, adhesions	ULAR	Ileostomy, SPC, Adhesiolysis	Coloanal hand
3	M	65	30	AH, DM		Rectal cancer at 2 cm, after TNT	APR	SPC	Descendostomy
4	M	57	21	AH, COPD	Bilateral TAPP	Diverticulitis, Adhesions	SR	AE, Adhesiolysis	31 CEEA at 9 cm
5	M	67	33	DM, AF, Hodgkin		Rectal cancer at 12 cm	LAR	AE, SPC	33 CEEA at 7 cm
6	M	68	23	AH, MI, CAD, DM, COPD		Sigmoid cancer at 20 cm + 3 polyps in descending colon	LH	AE	33 CEEA at 12 cm
7	F	57	21	AH	Open AE, lap OE	Diverticulitis, Adhesions	SR/LAR		Hand at 7 cm
8	F	74	22		Open AE and CCE	Diverticulitis	SR	AE	33 CEEA at 11 cm
9	F	66	31		Open HE + AE, lap CCE	Rectal cancer at 5 cm after TNT, Adhesions	ULAR	AE, SPC, Ileostomy, Adhesiolysis	33 CEEA at 3 cm
10	M	56	28	AH, DM	Lap loop sigmostomy	Rectal cancer at 6 cm after TNT, Adhesions, stomal hernia	ULAR	AE, SPC, Ileostomy, Stomal hernia repair	33 CEEA at 3 cm
11	F	66	25		Lap CCE, open AE, open OE	Diverticulitis	SR		Hand at 14 cm
12	F	61	28	LC, RA, AH, DM, COPD	Lap HE, open AE, multiple CS	Ascending colon cancer, liver cirrhosis with ascites	RH	S4 liver resection	Hand extracorp
13	F	67	30	AH	Open AE, lap fundoplication	Diverticulitis, rectal prolapse	SR	Rectopexy	Hand at 11 cm
14	M	33	30		Open Hartmann	Diverticulitis, persistent abscess, adhesions, stomal hernia	RHP	Adhesiolysis, Stomal henia repair	33 CEEA at 12 cm
15	M	55	35	AH		Diverticulitis	SR		33 CEEA at 12 cm
16	F	77	21	AH, Pericarditis	Open HE, open AE	Diverticulitis, Adhesions, rectal prolapse	SR	Adhesiolysis, Rectopexy	Hand at 14 cm
17	M	58	25			Rectal cancer at 11 cm	LAR	AE, SPC, Ileostomy	33 CEEA at 7 cm
18	F	72	25		Open HE + OE, open AE	Locally advanced caecal cancer,, hepatic metastasis S5	RH	CCE, Adhesiolysis, S5 metastasectomy	Hand, conversion
19	F	87	27	AH, CAD	Open AE, open CCE	Caecal cancer, invading right ovary and abdominal wall	RH	Right adnexectomy, Adhesiolysis	Endo-GIA 60
20	F	83	21	AH, AF, DM, long Covid	Open HE, lap AE	Colon cancer at hepatic flexure, cholecystitis, adhesions	RH	CCE, Adhesiolysis	Hand extracorp
21	M	67	31	AH	Prostatectomy, open AE	Diverticulitis	SR		Hand at 15 cm
22	M	41	17	MG, Cachexia		Rectal cancer at 7 cm after TNT, loop Sigmostomy	ULAR	AE, SPC, Ileostomy	31 CEEA at 3 cm
23	F	81	22		Open HE	Rectal cancer in anal canal, after 5 × 5 Gy radiation	ULAR	AE, SPC, Ileostomy	Coloanal hand
24	F	61	24			Diverticulitis	SR	AE	Hand at 13 cm
25	F	67	24	AH, CAD, RA	Open Hartmann, open AE	Diverticulitis, stump 5 cm, transversostomy, adhesions	RHP	Adhesiolysis, Ileostomy	33 CEEA at 3 cm

*SR* sigmoid resection, *LAR* low anterior resection, *ULAR* ultra-low anterior resection, *APR* abdomino-perineal resection, *RHP* reversal of Hartmann’s procedure, *RH* right hemicolectomy, *LH* left hemicolectomy, *SPC* suprapubic catheter, *AE* appendectomy, *CCE* cholecystectomy, *OE* ovarectomy, *HE* hysterectomy, *TNT* total neoadjuvant therapy, *CS* caesarean section, *AH* arterial hypertension, *DM* diabetes mellitus, *COPD* chronic obstructive pulmonary disease, *AF* atrial fibrillation, *MI* myocardial infarction, *CAD* coronary artery diasease, *LC* liver cirrhosis, *RA* rheumatoid arthritis, *MG* myasthenia gravis

**Table 2:** Duration of surgery, estimated blood loss, complications and hospital stay

Nr	Docking time (min)	Console time (min)	Total OP time (min)	EBL (ml)	Intraoperative difficulties	Postoperative complications	Stay [days]
1	18	120	220	150			6
2	15	227	322	150	Insufficient CEEA anastomosis, switch to coloanal	High output stoma, pneumonia, delirium	34
3	15	240	360	100			13
4	13	155	240	50			6
5	15	220	325	50			8
6	12	155	230	50			6
7	12	90	175	100	Lumen too small for CEEA, switch to hand anastomosis		8
8	12	115	200	< 50			8
9	12	240	375	200			10
10	15	352	420	200		Paralytic ileus	13
11	10	153	201	< 50			7
12	15	170	243	150	Flexure not accessible due to abdominal obesity and ascites		6
13	10	70	125	< 50		CT-Drain for late presacral hematoma	11
14	15	120	270	100	Rectal laceration by CEEA, lower re-resection		7
15	10	183	242	< 50		Transient compartment right calf	7
16	8	99	166	< 50			7
17	8	181	319	200		CT-Drain for late presacral hematoma	7
18	14	85	307	200	Late conversion due to severe adhesions and liver metastasis	Clostridial infection	14
19	12	346	422	100			6
20	10	253	317	150	90 min adhesiolysis, switch to open anastomosis		8
21	8	171	249	< 50	Lumen too small for CEEA, switch to hand anastomosis		7
22	8	219	326	100	Vulnerable tissue due to cortison	Anastomotic tear dorsally—EndoVAC therapy	16
23	8	250	317	< 50			9
24	7	91	138	< 50			7
25	11	168	320	200			9

*EBL* estimated blood loss, *CEEA* circular end-to-end anastomosis, *CT* computed tomography

A total of 14 female and 11 male patients underwent ten sigmoid resections, seven low anterior rectal resections, four right and one left hemicolectomies, two Hartmann’s procedure reversals, and one abdominoperineal resection. The median age was 66 years and the median body mass index 26 kg/m^2^. The most common indication for surgery was colorectal cancer (*n* = 13), followed by chronic recurring diverticulitis (*n* = 10) and two patients needing a reversal of a Hartmann’s procedure due to perforated diverticulitis in the past. Seventeen patients had previous history of abdominal surgery causing relevant extent of adhesions in ten of them. A significant proportion of patients (17/25, 68%) suffered relevant comorbidities, seven patients (28%) were on anticoagulation, and four (16%) were on life-long steroid therapy.

### Technical performance of the device

We experienced one technical problem with the device in the beginning of this series. In patient Nr. 3, one of the arms repeatedly did not recognize the instruments. The system had to be restarted twice until function was regained. The arm was successfully repaired before the next procedure. Also, a software update was necessary after the fifth procedure to eliminate some bugs. These technical problems led to a time delay, but no adverse clinical events. Subtle arm collisions were repeatedly detected, but tolerated by the device and did not interrupt proper function of the system. Neither instruments nor other hardware parts broke or showed defects. There were no problems with image or video transfer. There were no device-related patient injuries.

### Sigmoidectomy and left hemicolectomy

**Fig. 1:**
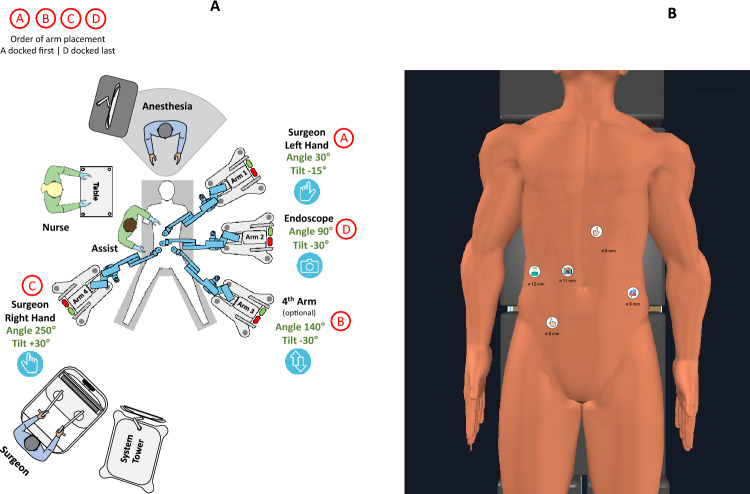
Left hemicolectomy and sigmoidectomy: cart setup (**A**) and trocar position (**B**)

Ten cases of sigmoidectomy for diverticulitis and one left hemicolectomy for cancer were performed based on the setup first proposed by Bianchi (Fig. 1) [8]. In seven patients, mobilization of the left colonic flexure was performed: in two of them fully robotic and in the rest combined robotic/laparoscopic. In four patients, a simultaneous robotic appendectomy was performed, in three cases a time-consuming robotic adhesiolysis was necessary. In two elderly female patients, the coexistent rectal prolapse was treated by a Frykman–Goldberg rectopexy. A 6 cm Pfannenstiel incision was used for specimen extraction. A descendorectostomy was performed at a median of 12 cm from the anal verge. A CEEA stapler was used in five patients, the rest received a two-layer continuous PDS 4-0 hand-sewn anastomosis: both patients with rectopexy, three others because of a narrow intestinal lumen not suitable for a stapler and in a patient with a kinking of the rectum, which made correct insertion of the stapler impossible. Docking time improved from 18 to 7 min over time. Median console time was 120 min. Median total operative time was 201 min. Median postoperative stay was 7 days. No conversions and no severe postoperative complications occurred: a female patient with a concomitant rectopexy was readmitted 3 weeks after surgery with pain in the lower abdomen and slightly elevated CRP—a 5 cm presacral haematoma was drained under CT guidance and antibiotics applied. An overweight male patient suffered in the early postoperative period a beginning of self-limited mild compartment syndrome of the lateral compartment of the right calf, which rapidly improved with mobilization.

### Low rectal resections (LAR and APR)

**Fig. 2:**
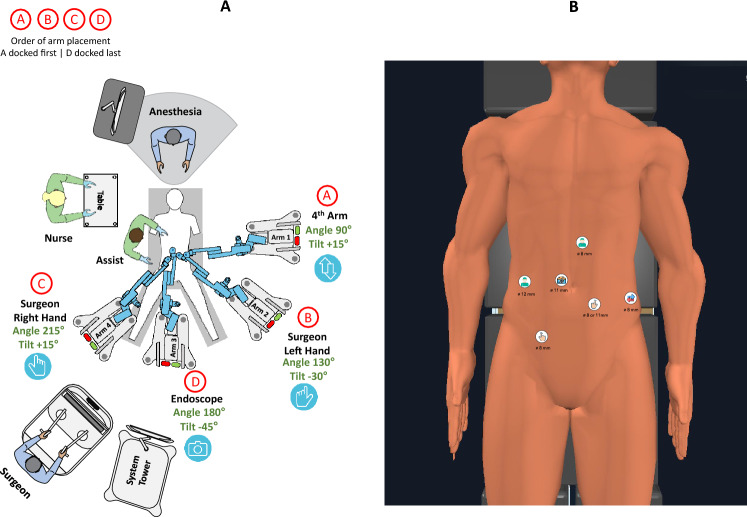
Rectal resection, APR and Hartmann’s reversal: cart setup (**A**) and trocar position (**B**)

The initial setup was the same as for sigmoid resection. This allowed vascular control of inferior mesenteric vessels as well as medial-to-lateral mobilization of the left colon. However, the port in the upper abdomen was usually far away from the pelvis. For adequate TME dissection down to the pelvic floor, two ports in the left lower abdomen were necessary and redocking performed. The fourth arm remained at its position, while the first three arms were rotated about 45° clockwise, so that the camera was attached to arm 3 between the legs, and arms 1 and 2 were used for the bipolar and the double fenestrated grasper (Fig. 2). The upper abdomen port could then be used to apply upward traction of the rectum, the assistant port for suction, irrigation, additional traction if needed and for stapled transection of the rectum. The two robotic hands allowed traction und countertraction and the dissection itself was done with the shears. In LAR, specimen extraction and insertion of the CEEA-anvil in the colon were performed via a 6 cm Pfannenstiel incision. In patients who underwent LAR and had already had a diverting sigmoidostomy, this step was done through the stomal opening. In APR and in LAR with a coloanal hand anastomosis, the specimen was extracted through the perineal wound or through the anus.

Eight patients underwent oncologic resections of the rectum for cancer, seven anterior resections and one abdominoperineal rectal extirpation. Tumors were located at a median height of 5 cm from the anal verge. In five patients, a total neoadjuvant chemoradiotherapy had been performed before surgery. One patient denied chemotherapy and had only short-term radiotherapy. Two patients with tumors in the upper third of the rectum did not receive neoadjuvant therapy. In two cases of ultralow anterior resection, a coloanal anastomosis was necessary, and the rest had stapled anastomosis. A loop ileostomy was diverted as standard in all but one of the patients. Four patients developed postoperative complications which resulted in a median postoperative stay of 11 days. Docking time improved from 15 to 8 min. Median console time was 241 min, while median total operative time was 345 min.

### Hartmann’s reversal

In candidates for a Hartmann’s reversal, colostomy was closed, and colon repositioned into the abdomen. The defect was temporarily closed with a small Alexis wound protector and covered with its cap, with one of the left-sided lower abdominal ports inserted through its opening. The port positioning was the same as for LAR. Resected parts of the colon and the rectal stump were extracted through the stomal defect, which was used also for insertion of the CEEA anvil in the colon. We performed two Hartmann reversals with a median console time of 145 min and median total operative time of 295 min. Both patients recovered uneventfully.

### Right hemicolectomy

**Fig. 3:**
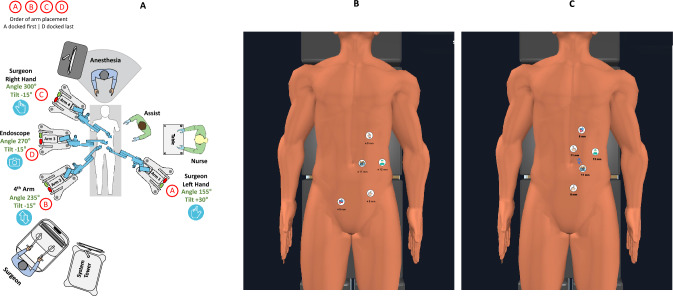
Right hemicolectomy: cart setup (**A**), standard trocar position (**B**) and alternative trocar position (**C**). *C* camera trocar, *A* assistant trocar, *LH* left hand of surgeon, *RH* right hand of surgeon, *R* reserve hand

There are two possible, basically different setup configurations for a right hemicolectomy. Our preferred setup was close to that one first reported by Bianchi, with three arms to the right of the patient and one arm at the left hip, connected to three ports in the left hemiabdomen and a port in the right iliac fossa (Fig. 3A and B) [8]. We also used once a modification of the vendor’s setup with a Z-formed port distribution directly to the left of the midline and a butterfly cart positioning; however, we suffered more collisions with it and there was less place and freedom for the bedside assistant (Fig. 3C).

None of the described setups was satisfactory and we are still in the process of developing our own setup for right hemicolectomy.

Docking time improved from 15 to 10 min. Median console time was 211 min, and median total operative time was 312 min. The long duration of surgery was partially caused by locally advanced tumours with infiltration of adjacent structures, lengthy adhesiolysis and additional pathology such as cirrhosis, liver metastasis or acute cholecystitis. Postoperative recovery was uneventful except for a clostridial infection in one of the patients, treated with oral antibiotics.

### Concomitant procedures performed with Hugo RAS during colorectal surgery

A partial or complete takedown of adhesions was robotically performed in ten patients. In two cases, serosal defects occurred which were immediately robotically oversewn. In ten patients a robotic appendectomy was performed, and in the other two a robotic cholecystectomy. Diversion of loop ileostomy was robotically assisted in seven cases.

### Systematic review of the literature

A systematic search in PubMed found 80 publications on the clinical use of Hugo™ RAS till April 20th, 2024. Only five of them reported data on a total of 23 colorectal resections [7–11]. Three Italian and two Spanish teams presented case series of one to nine patients. There were nine right and one left hemicolectomies, five ileocaecal, four rectal and four sigmoid resections. The summary of data is presented in Table 3. Port positioning, angles and tilts of arm carts described were not standardized and varied according to authors’ preferences and type of procedure; however, overall setups largely corresponded to our own shown in Figs. 1, 2, 3. Gender distribution (14 male and 9 female), median age of 66 and BMI of 26 kg/m^2^ did not significantly differ from those in our group. Median docking time was 10 min, median console time was 194 min and median total duration of surgery was 247 min. Median hospital stay was 5 days. No major technical problems with the device and no postoperative complications were reported.

**Table 3:** Summary of published case series on colorectal resections with Hugo™ RAS

Authors	Center	Year	Surgery	Diagnosis	Sex	Age (years)	BMI (kg/m^2^)	Dock time (min)	Console time (min)	OP time (min)	Stay [days]
Bianchi et al. [8]	Italy—Milan	2023	Left colectomy	Cancer	M	75	30	6	260	340	6
			Right colectomy	Cancer	F	66	27	10	255	336	4
			Right colectomy	Cancer	F	74	16	9	264	365	5
Gangemi et al. [7]	Italy—Bologna	2023	ICR	Diverticulitis	M	69	22	8	78	132	12
			ICR	Crohn	M	25	19	7	140	166	9
			ICR	Crohn	M	34	25	4	100	135	5
			ICR	Crohn	F	17	16	7	160	205	7
			ICR + CCE	Crohn	F	60	28	7	194	325	7
			Right colectomy	Cancer	M	83	31	7	170	235	10
			Sigmoidectomy	Diverticulitis	M	62	25	5	147	267	7
Caruso et al. [9]	Spain—Madrid	2023	Right colectomy	Cancer	M	–	20	5	–	200	8
Romero-Marcos et al. [10]	Spain—Barcelona	2024	Right colectomy	Cancer	M	59	28	10	–	180	3
			Right colectomy	Cancer	M	68	26	18	–	260	3
			Right colectomy	Cancer	F	62	34	14	–	240	3
			Right colectomy	Cancer	M	76	27	15	–	190	3
			Right colectomy	Cancer	F	71	26	16	–	170	3
			High rectum res	Cancer	F	72	29	10	–	200	3
			Sigmoidectomy	Diverticulitis	M	51	24	19	–	180	3
			Sigmoidectomy	Diverticulitis	F	71	31	13	–	160	3
			Sigmoidectomy	Cancer	M	65	25	14	–	150	4
Caputo et al. [11]	Italy—Rome	2024	High rectum res	Cancer	M	83	26	15	345	466	8
			High rectum res	Cancer	F	57	28	12	271	345	9
			Low rectum res	Cancer	M	72	20	8	397	435	7

*ICR* ileocolic resection, *CCE* cholecystectomy, *M* male, *F* female

## Discussion

In this study, we describe our early experience with colorectal surgery, performed with the use of the Hugo™ RAS system.

The advantages of Hugo lie in its innate robotic features—minimally invasive access, stable high-resolution 3D imaging with magnification, precise tireless tremor-free wristed movements in small space, rapid swap between bipolar and monopolar energy, ergonomic position and flexibility of the surgeon. Additionally, its open architecture improves team interaction and its modular concept with four individual arms allows configuration freedom, taking into account both surgeon’s preferences and patient’s characteristics. The pistol-like grips are very similar to those of laparoscopic instruments and thus comfortable for laparoscopic surgeons without previous experience in robotic surgery.

Limitations of the Hugo platform with respect to major colorectal surgery include the lack of a wristed robotic advanced energy device and some other relevant instruments, such as irrigation/suction, clip applicator, and linear stapler. In our experience, the 30° camera did not offer a great advantage compared to the 0° and was a source of more collisions. Lack of procedural setup memory and the modular design demand greater device-specific experience from the surgical team. Currently, the Hugo platform is still not widely distributed which results in shortage of experienced proctors. Thus, every Hugo surgeon nowadays has to invest much pioneering effort to pave the way for future users of the system.

A special feature of this study is the lack of patient selection. Opposite to most other reports on the early experience with Hugo, our cohort of patients represents consecutive cases, scheduled for surgery irrespective of their age, BMI, diagnosis, comorbidities or previous surgery. The only prerequisites for inclusion were signed informed consent for Hugo surgery, availability of the robotic surgical team and presence of a start-up specialist. The limited number of procedures reflects initial shortage of instrument supply and the temporary stop due to regulatory issues in Germany. An unprecedentedly large portion of our patients had previous surgery resulting in relevant adhesions. Also, concomitant surgical pathology and comorbidities were significantly more prevalent than in the five other cases series. Time-consuming adhesiolysis and additional procedures such as cholecystectomy, appendectomy or rectopexy partially explain the long duration of surgery. The last could also be affected by the lack of previous robotic experience of the team. However, previous experience with da Vinci does not directly transfer into shorter operative times or better outcomes when using Hugo, as shown by the results of Romero-Marcos et al. and Caputo et al. [10, 11]. The console part of surgery is very similar between Hugo and da Vinci. However, positioning of the trocars and configuration of the arms require a completely different approach, making previous da Vinci experience of little advantage to novice Hugo users. The absence of experienced proctors and practically proven established setup guides have undoubtedly affected our docking, console and total operative times, especially at the beginning of the series.

Regarding docking time, ours averaged 12 min and was thus somewhat longer than reported by other authors. On the one hand, procedure-specific docking times improved in every subgroup over time. On the other hand, correct docking is of utmost importance to avoid later collisions ensuring optimal volume of instrument movement. As optimal docking can save a lot of console time, we prefer to invest some more minutes and patience proving the volume of movement and testing the setup rather than pursuing fast docking and then suffer later poor access, annoying collisions, and lack of mobility.

Setup configuration of the arm carts varies between different authors and the ideal setup for every procedure is still to be found. Regarding left and right hemicolectomy, we adopted from the very beginning the setup proposed by Bianchi et al. with three arms on one side and the fourth arm at the opposite hip of the patient, which was also applied by Romero-Marcos and Caruso [8–10]. This configuration allows collision-free medial to lateral dissection combined with early vessel control and offers more working space and safety to the bedside assistant. Other teams, such as those of Caputo and Gangemi, preferred the universal butterfly setup, originally proposed by Medtronic with two carts on each side of the patient [7, 11]. For complete TME deep in the pelvis with or without anastomosis, the modified Medtronic setup with the optic arm between the legs, one port in the right lower abdomen, and two ports in the left lower abdomen proved to be most appropriate and seems to be accepted by all authors.

Concerning instrument selection for colorectal procedures, we prefer using a double-fenestrated grasper rather than a cadiere, because of its larger jaws offering better retraction especially during TME dissection and because of its gentle atraumatic touch. Both graspers however possess a very low grasping force, which makes manual cranial retraction of the rectum during LAR a preferred option, especially in obese patients. Due to its sharp jaws with small coagulating surface, we avoid the use of the Maryland grasper.

In our experience, right hemicolectomy turned out to be the most challenging procedure. Irrespective of trocar and cart positioning, mobilization of the hepatic flexure and division of the greater omentum and the transverse mesocolon were time consuming, inconvenient and to a certain extent even risky. The lack of a robotically driven advanced energy device was the main reason—it compelled the bedside assistant to use a laparoscopic Ligasure through the assistant 12 mm port and the console surgeon was restricted to use two arms for traction and exposing anatomy. Depending on the body structure of the patient, especially in those tall and/or overweight, either the arm in the left or the arm in the right lower abdomen did not sufficiently reach the region of interest. Furthermore, the 0° camera sometimes offers insufficient view of the hepatic flexure. The 30° camera improved the view, but at the cost of increased collisions. In our opinion, Hugo RAS is currently feasible and safe to perform ileocoecal resection and standard right hemicolectomy mainly for benign findings or small malignant tumors in the caecum or proximal ascending colon. Extended oncologic complete mesocolic excision (CME) with a D3 lymph node dissection along the vessel wall should rather wait until the introduction of the wristed Hugo-Ligasure and upgraded 30° camera. We had the chance to test two working prototypes (straight and wristed ones) in a wet laboratory at ORSI, Melle, already a year ago. However, certification and market introduction are expected by early 2025.

In conclusion, according to our initial experience, Hugo RAS can be successfully used to perform major colorectal surgery. Its open modular design offers flexibility and individual adjustments, as well as the high-quality magnified 3D imaging offers advantages in performing complex procedures and makes the platform an effective alternative to other established robotic systems. The continuous software updates together with the much-awaited introduction of an expanded palette of instruments such as a wristed vessel sealing device and improvements in the camera system will make Hugo RAS even more user-friendly and thus increase its use for complex procedures in general surgery.

## Data Availability

All raw data analysed in this article are available and saved on the data server of Katholisches Klinikum Bochum. Access may be requested at any time by addressing the Ethic Committee of the Ruhr University Bochum.
